# circ_0000467 promotes the proliferation, metastasis, and angiogenesis in colorectal cancer cells through regulating KLF12 expression by sponging miR-4766-5p

**DOI:** 10.1515/med-2021-0358

**Published:** 2021-09-24

**Authors:** Hui Chen, Chen Wu, Liang Luo, Yuan Wang, Fangxing Peng

**Affiliations:** Department of Gastroenterology and General Surgery, Sichuan Mianyang 404 Hospital, 621000, Mianyang, Sichuan, China; Department of Pediatric Infectious Diseases, Sichuan Mianyang 404 Hospital, 621000, Mianyang, China; Department of Gastroenterology and General Surgery, Sichuan Mianyang 404 Hospital, No. 56, Yuejin Street, Fucheng District, 621000, Mianyang, Sichuan, China

**Keywords:** colorectal cancer, circ_0000467, miR-4766-5p, KLF12

## Abstract

**Background:**

Circular RNAs have been identified as crucial players in the initiation and progression of cancers, including colorectal cancer (CRC). The Has_circ_0000467 (circ_0000467) expression has been found to be upregulated in CRC, but its function and mechanism remain unclear.

**Methods:**

The expression levels of circ_0000467, microRNA-4766-5p (miR-4766-5p), and Krueppel-like factor 12 (KLF12) were examined using reverse transcription-quantitative polymerase chain reaction. Cell proliferation was analyzed by cell counting kit-8 assay and colony formation assay. The apoptosis was measured by flow cytometry. Transwell migration and invasion assays were applied to evaluate cell metastatic ability. Angiogenesis was detected using tube formation assay. All protein expressions were quantified by western blot assay. Dual-luciferase reporter assay was used to analyze intergenic binding. Xenograft models were constructed for the experiment of circ_0000467 *in vivo*.

**Results:**

The expression of circ_0000467 was upregulated in CRC tissues and cells. Knockdown of circ_0000467 repressed cell proliferation, metastasis, and angiogenesis, but it induced apoptosis in CRC cells. circ_0000467 targeted miR-4766-5p and inhibited the expression of miR-4766-5p. Silencing of circ_0000467 inhibited CRC progression by upregulating miR-4766-5p. miR-4766-5p suppressed the expression of target gene KLF12 and KLF12 overexpression reversed the effects of miR-4766-5p on CRC cell behaviors. circ_0000467 positively regulated the expression of KLF12 by targeting miR-4766-5p. circ_0000467 downregulation *in vivo* reduced CRC tumorigenesis by regulating miR-4766-5p and KLF12.

**Conclusion:**

circ_0000467 acted as an oncogene in CRC through regulating KLF12 expression by sponging miR-4766-5p. Therefore, circ_0000467 can be used as an effective target in CRC diagnosis and therapy.

## 1 Introduction

Colorectal cancer (CRC) is one of the most common malignant tumors and the incidence rate is increasing with age [1,2]. There are 1–2 million patients who have been diagnosed with CRC, and the mortality is higher than 50% [2]. Improving early diagnosis and preventing late treatment may be crucial for CRC patients. Growing molecular markers have been reported to be related to the prediction and prognosis of CRC [3]. More diagnostic and therapeutic targets have to be discovered.

Circular RNAs (circRNAs) are endogenous non-coding RNAs in mammalian cells, and usually function as sponges of microRNAs (miRNAs) to regulate cellular processes in cancers [4]. In CRC, circ_0137008 inhibited the malignant phenotype by acting as a molecular miR-338-5p sponge [5]; circHUWE1 enhanced cell proliferation and metastasis by sponging miR-486 [6]. Hsa_circ_0000467 (circ_0000467) has been proved to be a cancer-promoting molecule in gastric cancer (GC) via the sponge effect on miR-326-3p [7]. The function of circ_0000467 in CRC was researched in this study. MicroRNA-4766-5p (miR-4766-5p) was a tumor inhibitor in GC [8] and CRC [9]. However, the potential of circ_0000467 to act as a sponge for miR-4766-5pin CRC remains unclear.

miRNAs affect gene expression to regulate cancer progression by binding to the 3′-untranslated regions (3′-UTRs) of precursor mRNAs [10,11]. For instance, miR-4766-5p impeded the development of breast cancer by downregulating the expression of SIRT1 [12]. Krueppel-like factor 12 (KLF12) is a well-known oncogene in different kinds of human cancers such as pancreatic cancer [13], gastric cancer [14], and endometrial cancer [15]. KLF12 was upregulated in CRC and cell growth was also facilitated by the overexpression of KLF12 [16]. The assumption that miR-4766-5p targeted KLF12 was explored in this study.

The circRNA/miRNA/mRNA regulatory network has been considered as the important functional mechanism in CRC [17]. In addition to the biological role of circ_0000467 in CRC, we also focused on the relationship axis of circ_0000467 with miR-4766-5p and KLF12.

## 2 Materials and methods

### 2.1 Specimen acquisition and cell culture

CRC tissue samples (*n* = 30) and the adjacent normal counterparts (*n* = 30) were collected from CRC patients in Sichuan Mianyang 404 Hospital. These samples were preserved in liquid nitrogen before the extraction of total RNA or protein. All patients signed the written informed consent form for this research. This study was authorized by the Ethics Committee of Sichuan Mianyang 404 Hospital and was conducted as per the guidelines of the Declaration of Helsinki.

Human CRC cell lines (LOVO and HCT116) were purchased from the American Type Culture Collection (ATCC, Manassas, VA, USA) for our CRC research and the human colon mucosal epithelial cell line NCM460 was applied as the normal control. These cells were cultured in 5% CO_2_ at 37°C using RPMI-1640 medium containing 10% fetal bovine serum (FBS; GIBCO, Carlsbad, CA, USA) and 1% penicillin–streptomycin solution (GIBCO).

### 2.2 Cell transfection

The small-interfering RNA for circ_0000467 (si-circ_0000467), short hairpin RNA vector for circ_0000467 (sh-circ_0000467), miR-4766-5p inhibitor or mimic (anti-miR-4766-5p or miR-4766-5p), and the corresponding negative controls (si-NC, sh-NC, anti-miR-NC, and miR-NC) were acquired from RIBOBIO (Guangzhou, China). The pCE-RB-Mam-circ_0000467 (circ_0000467) and pcDNA-KLF12 (KLF12) overexpression vectors were generated by cloning their sequences into the basic vectors pCE-RB-Mam (RIBIBIO) and pcDNA (Invitrogen, Carlsbad, CA, USA), respectively. Lipofectamine 3000 (Invitrogen) was applied to perform cell transfection, according to the operating procedures of the producer.

### 2.3 Reverse transcription-quantitative polymerase chain reaction (RT-qPCR)

RNA was isolated from tissue samples and cells using Trizol reagent (Invitrogen) and reversely transcribed into the complementary DNA (cDNA) by Prime Script TM RT reagent kit (Takara, Shiga, Japan), complying with the manufacturer’s directions. The RT-qPCR was performed by SYBR^®^ Premix Ex Taq™ II Kit (Takara) on ABI Step One Real-time PCR System (Thermo Fisher Scientific, Waltham, MA, USA). Data were analyzed by the 2^−∆∆Ct^ method, using glyceraldehyde-3-phosphate dehydrogenase (GAPDH; for circ_0000467 and KLF12) and U6 (for miR-4766-5p) as the reference genes. Forward (F) and reverse (R) primers used in the study are shown as follows: circ_0000467 (F: 5′-ACACAATGGGACTTAAAAATGCGA-3′ and R: 5′-ACAGATCATCTTTCACATCAGTCT-3′); miR-4766-5p (F: 5′-GCCGAGTCTGAAAGAGCAGTT-3′ and R: 5′-CAGTGCAGGGTCCGAGGTAT-3′); KLF12 (F: 5′-CACCTGGAAATGTGAACAACA-3′ and R: 5′-TTTTACTTTGTCTGGGAGATAGGC-3′); GAPDH (F: 5′-GGTCGGAGTCAACGGATTTG-3′ and R: 5′-ATGAGCCCCAGCCTTCTCCAT-3′); and U6 (F: 5′-CTCGCTTCGGCAGCACA-3′ and R: 5′-AACGCTTCACGAATTTGCGT-3′).

### 2.4 Cell counting kit-8 (CCK-8) assay

LOVO and HCT116 cells were seeded into the 96-well plates overnight. After transfection for different times (24, 48, and 72 h), 10 μL of CCK-8 (Beyotime, Shanghai, China) was used to incubate cells for 4 h. The absorbance at 450 nm was detected on a microplate reader (Thermo Fisher Scientific).

### 2.5 Colony formation assay

The 12-well plates were inoculated with LOVO and HCT116 cells of 200 cells/well. Then the cells were incubated at 37°C for 2 weeks. After cell fixation in methanol (Beyotime) and staining in crystal violet (Beyotime), the colony number was counted.

### 2.6 Flow cytometry

These collected LOVO and HCT116 cells were washed with pre-cooled PBS (GIBCO) and re-suspended in 1× binding buffer (BD Biosciences, San Diego, CA, USA). Cell resuspension was added with Annexin V-fluorescein isothiocyanate (Annexin V-FITC) and propidium iodide reagent (BD Biosciences), following the user’s guideline. Eventually, the stained apoptotic cells were determined by the flow cytometer (BD Biosciences).

### 2.7 Transwell migration and invasion assays

LOVO and HCT116 cells were suspended in serum-free RPMI-1640 medium. The upper chamber of transwell chamber (Corning Life Sciences, Corning, NY, USA) was then seeded with cell suspension, and a 10% FBS + RPMI-1640 medium was added into the lower chamber. After 24 h, the cells in the upper chamber which had passed through the membranes into the lower chamber were fixated and stained in methanol and crystal violet (Beyotime). Finally, the migrated and invaded cells were photographed and counted under a microscope (100× magnification; Olympus, Tokyo, Japan). The upper chamber needs to be pro-coated with matrigel (Corning Life Sciences) before seeding cells, especially for invasion detection.

### 2.8 Tube formation assay

A total of 5 × 10^4^ human umbilical vein endothelial cells (HUVECs, ATCC) were seeded into the 24-well plates pre-coated with ice-cold Matrigel (BD Biosciences). Conditioned medium was collected from LOVO and HCT116 cells after transfection, then added into the wells with HUVEC for 24 h. The tube-like structures were imaged on a microscope and angiogenesis was assessed by counting the tubule length.

### 2.9 Dual-luciferase reporter assay

Starbase 3.0 and Targetscan software were used for target prediction for circ_0000467 and miR-4766-5p. The sequences of wild-type circ_0000467 and KLF12 3′-UTR containing miR-4766-5p binding sites were, respectively, inserted into the pmirGLO luciferase reporter vector (Promega, Madison, WI, USA) to construct the luciferase reporter plasmids (WT-circ_0000467 and WT-KLF12). The mutant-type controls containing mutant miR-4766-5p-binding sites were named as MUT-circ_0000467 and MUT-KLF12. LOVO and HCT116 cells were co-transfected with WT-circ_0000467/MUT-circ_0000467 and miR-4766-5p/miR-NC or WT-KLF12/MUT-KLF12 and miR-4766-5p/miR-NC. Cells were then harvested using 1× Passive Lysis Buffer (Promega) and the relative luciferase activity was assayed with the dual-luciferase reporter system (Promega).

### 2.10 Western blot (WB) assay

Total protein was extracted and prevented from degrading by using RIPA lysis buffer with proteinase inhibitor phenylmethanesulfonyl fluoride (Pierce, Rockford, IL, USA). Around 35 μg of proteins in each group were separated by SDS-PAGE for 90 min, and the separated proteins were transferred on to polyvinylidene fluoride membranes (Thermo Fisher Scientific). After the non-specific binding was blocked by 5% nonfat milk, the membranes were incubated with primary antibodies anti-KLF12 (Abcam, Cambridge, United Kingdom; ab129459, 1:1,000), anti-cyclinD1 (Abcam; ab1663, 1:1,000), anti-cleaved-casp-3 (Abcam, ab2302, 1:1,000), anti-E-cadherin (Abcam, ab40772, 1:1,000), and anti-GAPDH (Abcam; ab181602, 1:1,000) overnight at 4°C. Following the incubation of the secondary antibody (Abcam; ab205718, 1:5,000) for 1 h at room temperature, the chromogenic reaction was conducted by using the enhanced chemiluminescence (ECL) kit (Bio-Rad, Hercules, CA, USA).

### 2.11 *In vivo* experiment

Male BALB/c nude mice (6-week-old, *n* = 10; Vital River Laboratory Animal Technology Co. Ltd, Beijing, China) were subcutaneously injected with 3 × 10^6^ HCT116 cells transfected with sh-NC or sh-circ_0000467 vector (5 mice/group). After cell injection for 1 week, tumor volume (length × width^2^ × 0.5) was measured every 4 days. Tumors were taken out from the sacrificed mice 27 days later and the weight was examined on an electronic scale. Then the determination of circ_0000467, miR-4766-5p, and KLF12 was performed by RT-qPCR or WB following the isolation of RNA and protein form tumor tissues. All mice were cared according to the National Institutes of Health Guide for the Care and Use of Laboratory Animals, and this assay was authorized by the Animal Ethical Committee of Sichuan Mianyang 404 Hospital.

### 2.12 Statistical analysis

Each experiment was repeated for three times in triplicate (*n* = 3) and the total data were shown as mean ± standard deviation. Statistical analysis was performed by SPSS 19.0. The linear relationships were analyzed using Pearson’s correlation coefficient. Student’s *t*-test and one-way analysis of variance were conducted to analyze the statistical difference. If *P* < 0.05, the difference was identified as significant.

## 3 Results

### 3.1 circ_0000467 was upregulated in CRC tissues and cells

**Figure 1:**
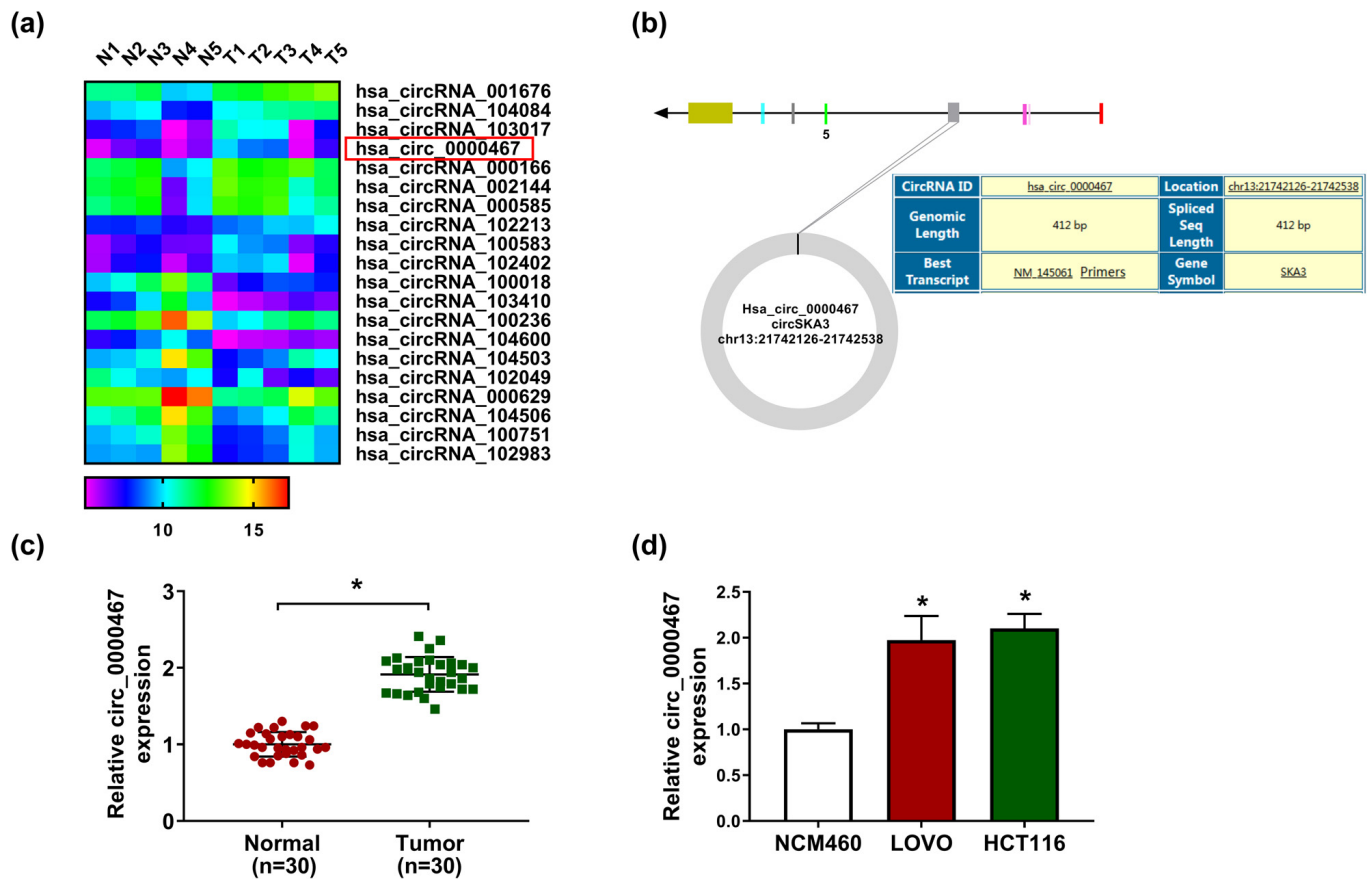
circ_0000467 was upregulated in CRC tissues and cells. (a) The top ten upregulated and downregulated circRNAs in CRC tissues were searched by GEO datasets. (b) The genic information of circ_0000467. (c and d) RT-qPCR was used to analyze circ_0000467 expression in CRC tissues (c) and cells (d). *P < 0.05.

According to the dysregulated circRNA analysis of GEO datasets, circ_0000467 was one of the upregulated circRNAs in CRC tissues (Figure 1a). circ_0000467 is an exonic circRNA from the spindle and kinetochore-associated complex subunit 3 (SKA3) gene with the spliced sequence length of 412 bp in the chr13: 21742126-21742538 (Figure 1b). In accordance with the data analysis, our RT-qPCR detection showed that the expression level of circ_0000467 had remarkably increased in 30 CRC tissues when compared with 30 normal tissues (Figure 1c). Also, circ_0000467 expression was much higher in LOVO and HCT116 cells than in normal NCM460 cells (Figure 1d). The dysregulation of circ_0000467 implied that circ_0000467 might play an important role in CRC.

### 3.2 Knockdown of circ_0000467 inhibited CRC cell proliferation, metastasis, and angiogenesis while promoted apoptosis

**Figure 2:**
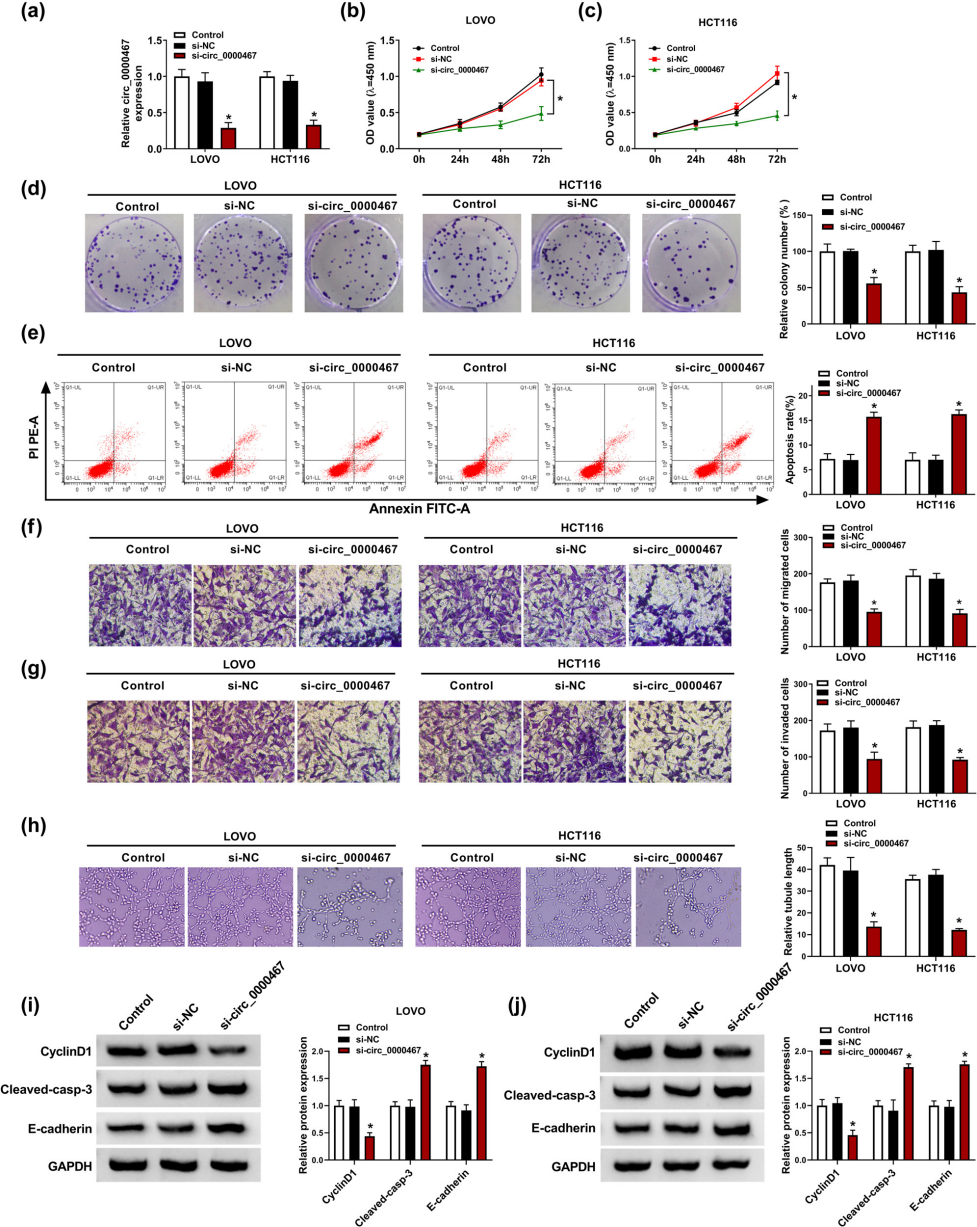
Knockdown of circ_0000467 inhibited CRC cell proliferation, migration, and invasion but promoted apoptosis. LOVO and HCT116 cells were transfected with si-circ_0000467 or si-NC, with untransfected cells as the control group. (a) The interfering effectiveness of si-circ_0000467 was detected by RT-qPCR. (b–d) Cell proliferation was examined by CCK-8 assay (b and c) and colony formation assay (d). (e) Cell apoptosis was analyzed by flow cytometry. (f and g) Cell metastasis was determined by transwell migration (f) or invasion (g) assay. (h) Angiogenesis was assessed by tube formation assay. (i and j) CyclinD1, cleaved-casp-3, and E-cadherin protein levels were assayed by WB. *P < 0.05.

To investigate the role of circ_0000467 in CRC progression, LOVO and HCT116 cells were transfected with si-circ_0000467 using si-NC as the negative control. The transfection of si-circ_0000467 reduced the circ_0000467 expression compared to the control group and si-NC group, showing that the interfering effect of si-circ_0000467 was significant (Figure 2a). Afterward, cellular behaviors were determined to assess the effect of circ_0000467 on CRC development. CCK-8 assay (Figure 2b and c) and colony formation assay (Figure 2d) presented that si-circ_0000467 transfection led to an inhibitory effect on cell proliferation, while flow cytometry showed an accelerative effect of si-circ_0000467 on the apoptotic rate (Figure 2e). By conducting transwell assay, we found that the migrated and invaded cells of the si-circ_0000467 group were much less than those in the control and si-NC groups (Figure 2f and g). Tube formation assay demonstrated that the silencing of circ_0000467 reduced angiogenesis in LOVO and HCT116 cells (Figure 2h). In addition, WB revealed that cyclinD1 protein expression was decreased while Cleaved-casp-3 and E-cadherin were upregulated after circ_0000467 downregulation (Figure 2i and j). These results showed that circ_0000467 knockdown inhibited the malignant process of CRC cells.

### 3.3 circ_0000467 functioned as a sponge of miR-4766-5p in CRC cells

**Figure 3:**
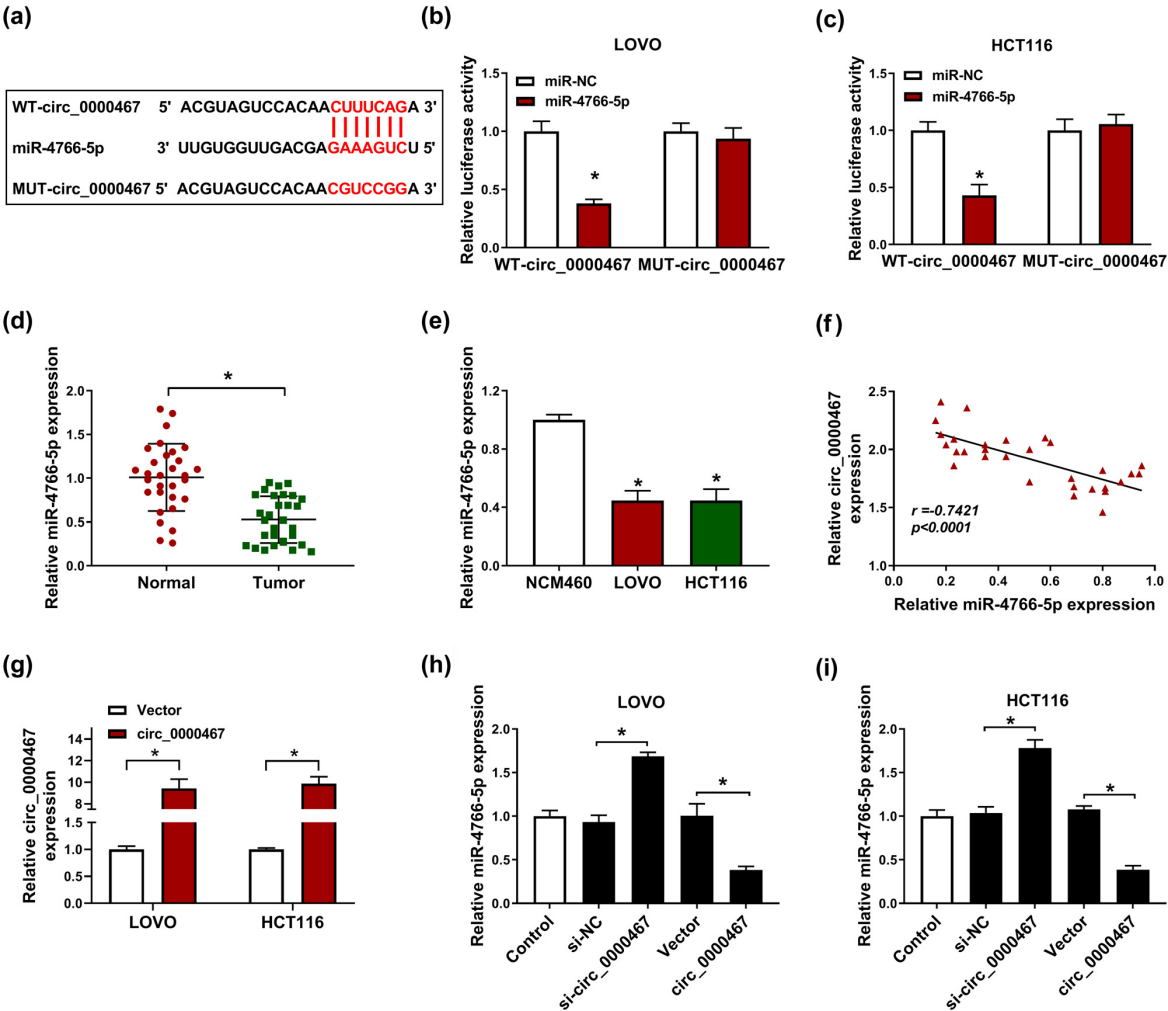
circ_0000467 functioned as a sponge of miR-4766-5p in CRC cells. (a) The presumptive binding sites between circ_0000467 and miR-4766-5p were predicted by Starbase. (b and c) The relative luciferase activity of LOVO and HCT116 cells co-transfected with WT-circ_0000467 or MUT-circ_0000467 and miR-4766-5p or miR-NC was measured by the dual-luciferase reporter system. (d and e) miR-4766-5p expression was assayed by RT-qPCR in CRC tissues (d) and cells (e). (f) The relationship between circ_0000467 and miR-4766-5p was analyzed with Pearson’s correlation coefficient. (g) The overexpression effect of circ_0000467 vector on circ_0000467 was detected through RT-qPCR. (h and i) The expression of miR-4766-5p was determined in LOVO and HCT116 cells with circ_0000467 overexpression or downregulation using RT-qPCR. *P < 0.05.

Starbase analysis indicated that the sequence of circ_0000467 contained the presumptive binding points of miR-4766-5p (Figure 3a). Dual-luciferase reporter detection was conducted after WT-circ_0000467 or MUT-circ_0000467 plasmid was transfected into LOVO and HCT116 cells with miR-4766-5p or miR-NC to explore the interaction between circ_0000467 and miR-4766-5p. As Figure 3b and c reveals, miR-4766-5p transfection reduced the relative luciferase activity of the WT-circ_0000467 group compared to miR-NC transfection, while the reduction was not noticed in the MUT-circ_0000467 group. The RT-qPCR results suggested that miR-4766-5p expression was significantly downregulated in CRC tissues (Figure 3d) and cells (LOVO and HCT116) (Figure 3e). The correlation between circ_0000467 and miR-4766-5p was found to be conspicuously negative (*r* = −0.7421, *P* < 0.0001) (Figure 3f). The circ_0000467 vector was used for overexpression of circ_0000467 and the transfection efficiency was found to be excellent (Figure 3g). Besides, the miR-4766-5p level was upregulated by the knockdown of circ_0000467 while circ_0000467 overexpression evoked the expression repression of miR-4766-5p (Figure 3h and i). circ_0000467 exerted the sponge effect on miR-4766-5p.

### 3.4 Downregulation of miR-4766-5p restored the si-circ_0000467-induced effect on CRC cells

**Figure 4:**
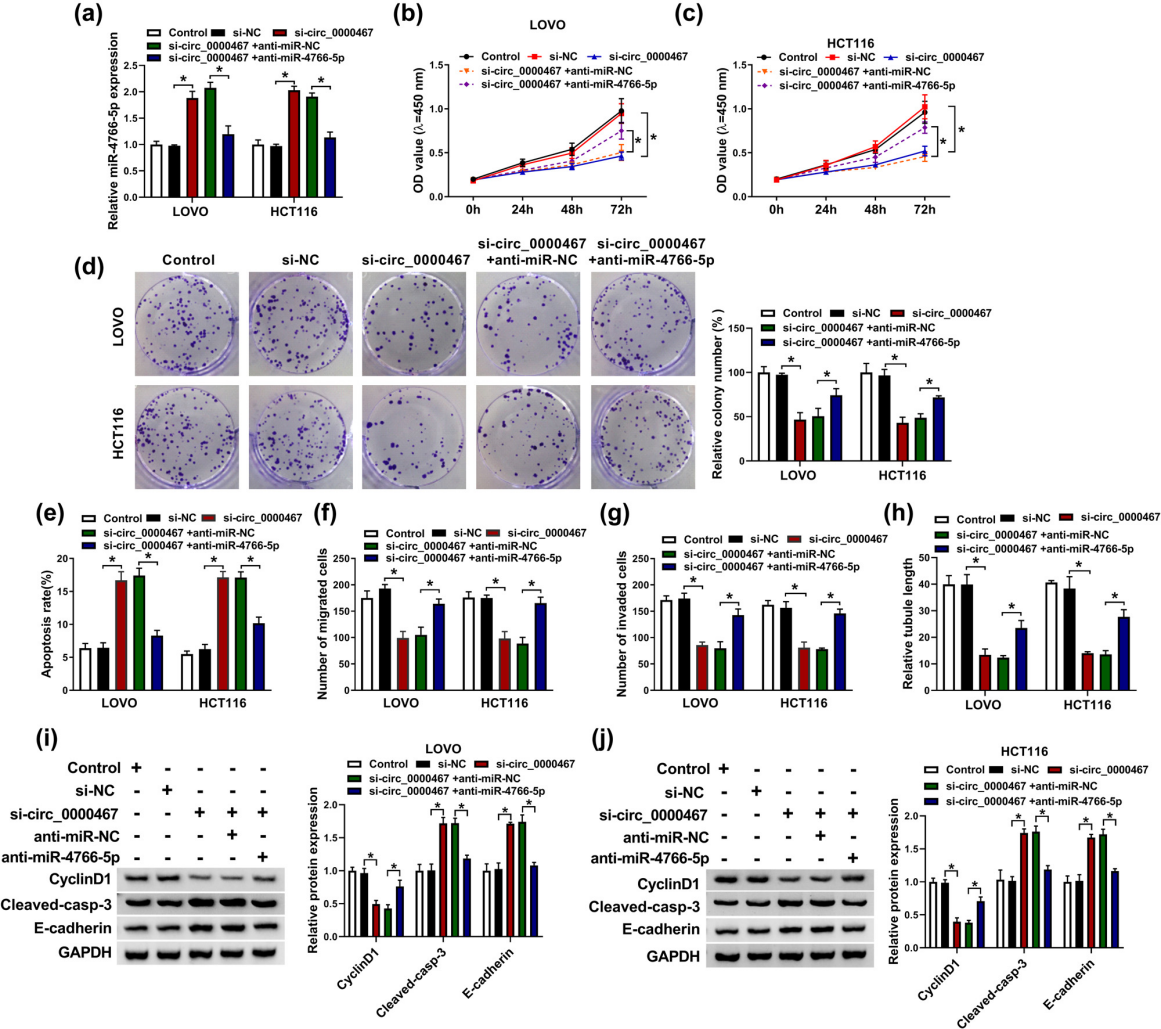
Downregulation of miR-4766-5p restored the si-circ_0000467-induced effect on CRC cells. (a) The RT-qPCR was used for miR-4766-5p detection after transfection of si-circ_0000467 and si-circ_0000467 + anti-miR-4766-5p or the matched controls. (b–d) The proliferative abilities of the above-transfected LOVO and HCT116 cells were detected with the CCK-8 assay (b and c) and colony formation assay (d). (e) Apoptotic rate was estimated through flow cytometry. (f and g) Transwell assay was performed to determine cell migratory (f) and invasive (g) abilities. (h) Tube formation assay was performed to measure the angiogenesis. (i and j) WB was used to quantify the protein expression of cyclinD1, cleaved-casp-3, and E-cadherin. *P < 0.05.

The rescued experiments were conducted to analyze the association of miR-4766-5p with the function of circ_0000467 in CRC. The anti-miR-4766-5p-mediated miR-4766-5p inhibition was great because it counteracted the promotive effect of si-circ_0000467 on miR-4766-5p (Figure 4a). With the downregulation of miR-4766-5p expression in LOVO and HCT116 cells, the si-circ_0000467-induced proliferation inhibition (Figure 4b–d) and apoptosis promotion (Figure 4e) were partly offset. The repressive effects on cell metastasis (Figure 4f and g) and angiogenesis (Figure 4h) caused by si-circ_0000467 were also abolished following the introduction of miR-4766-5p inhibitor. Similarly, the protein changes of si-circ_0000467 on cyclinD1, cleaved-casp-3, and E-cadherin were countervailed by the repression of miR-4766-5p (Figure 4i and j). Thus, the function of circ_0000467 was partly achieved by sponging miR-4766-5p in CRC.

### 3.5 miR-4766-5p targeted KLF12 in CRC cells

**Figure 5:**
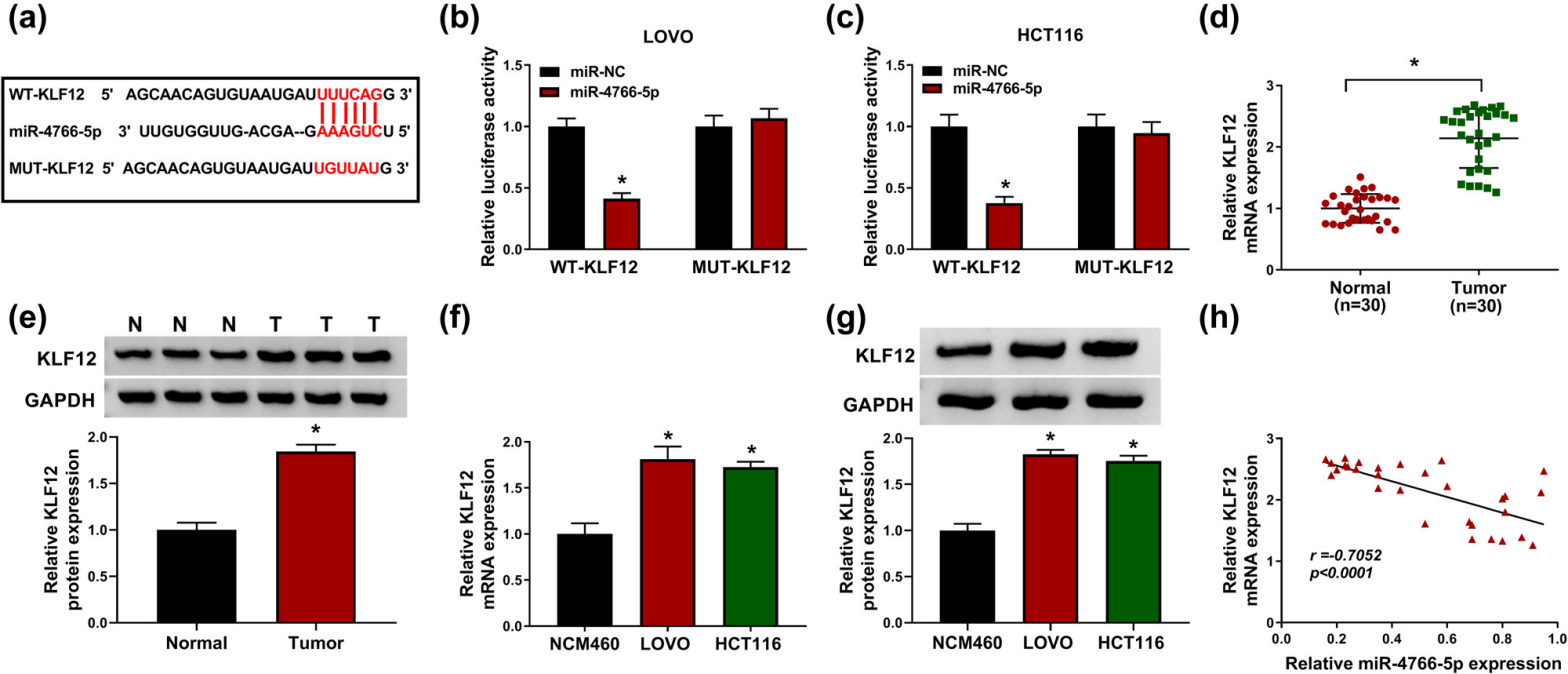
miR-4766-5p targeted KLF12 in CRC cells. (a) The binding of miR-4766-5p and KLF12 was predicted by Starbase. (b and c) Dual-luciferase reporter assay was used to prove the mutual effect of miR-4766-5p and KLF12. (d–g) The mRNA and protein levels of KLF12 were determined by RT-qPCR and WB in CRC tissues (d and e) and cells (f and g). (h) The correlation between miR-4766-5p and KLF12 was validated using Pearson’s correlation coefficient. *P < 0.05.

Starbase has predicted site binding between the 3′-UTR of KLF12 and miR-4766-5p sequences (Figure 5a). The dual-luciferase reporter assay revealed that overexpression of miR-4766-5p decreased the luciferase activity of LOVO and HCT116 cells transfected with WT-KLF12 not MUT-KLF12 (Figure 5b and c). KLF12 mRNA and protein expression levels were upregulated in CRC tissues (Figure 5d and e) and cells (Figure 5f and g). Linear analysis indicated a negative correlation (*r* = −0.7052, *P* < 0.0001) between miR-4766-5p and KLF12 levels in CRC tissues (Figure 5h). These data suggested that KLF12 was a downstream gene of miR-4766-5p in CRC cells.

### 3.6 miR-4766-5p played a suppressive role in the progression of CRC cells via downregulation of KLF12

**Figure 6:**
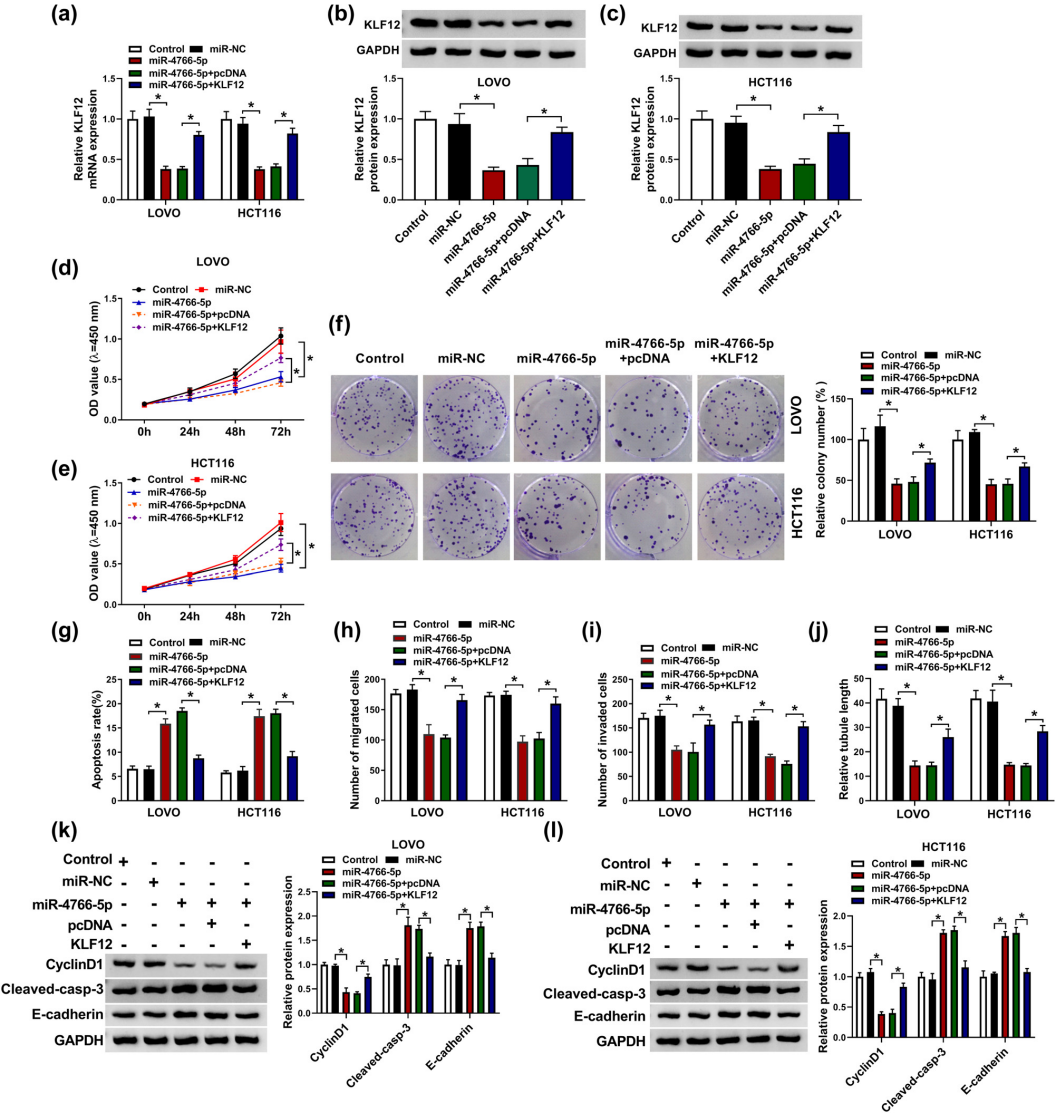
miR-4766-5p played a suppressive role in the progression of CRC cells via downregulation of KLF12. (a–c) KLF12 expression after transfection with miR-4766-5p, miR-4766-5p + KLF12 or their controls was assayed through RT-qPCR (a) and WB (b and c) in LOVO and HCT116 cells. (d–f) CCK-8 assay (d and e) and colony formation assay (f) were used for cell proliferation analysis. (g) Flow cytometry was used for cell apoptosis detection. (h and i) Cell metastatic abilities were evaluated by transwell assay for migration (h) and invasion (i). (j) The examination of angiogenesis was evaluated using tube formation assay. (k and l) Protein determination for cyclinD1, cleaved-casp-3, and E-cadherin was conducted using WB. *P < 0.05.

The involvement of KLF12 in the miR-4766-5p-mediated effect on CRC progression was studied after the transfection of miR-4766-5p and miR-4766-5p + KLF12 or relative negative groups. The introduction of miR-4766-5p prominently downregulated the mRNA (Figure 6a) and protein (Figure 6b and c) expression of KLF12, which was reversed by the overexpression of KLF12. Through the detection of CCK-8 and colony formation assays (Figure 6d–f), we found that miR-4766-5p mimic triggered the inhibition of proliferative ability, while KLF12 upregulation ameliorated this effect in LOVO and HCT116 cells. The apoptotic promotion caused by miR-4766-5p was also reversed after KLF12 expression was increased (Figure 6g). Additionally, the overexpression of KLF12 also abolished the inhibitory effects of miR-4766-5p on migration (Figure 6h), invasion (Figure 6i), and angiogenesis (Figure 6j). The same restoration of KLF12 for miR-4766-5p was found in the protein levels of cyclinD1, cleaved-casp-3, and E-cadherin (Figure 6k and l). Taken together, miR-4766-5p inhibited CRC progression via targeting KLF12.

### 3.7 Silencing circ_0000467 repressed KLF12 expression via upregulation of miR-4766-5p

**Figure 7:**
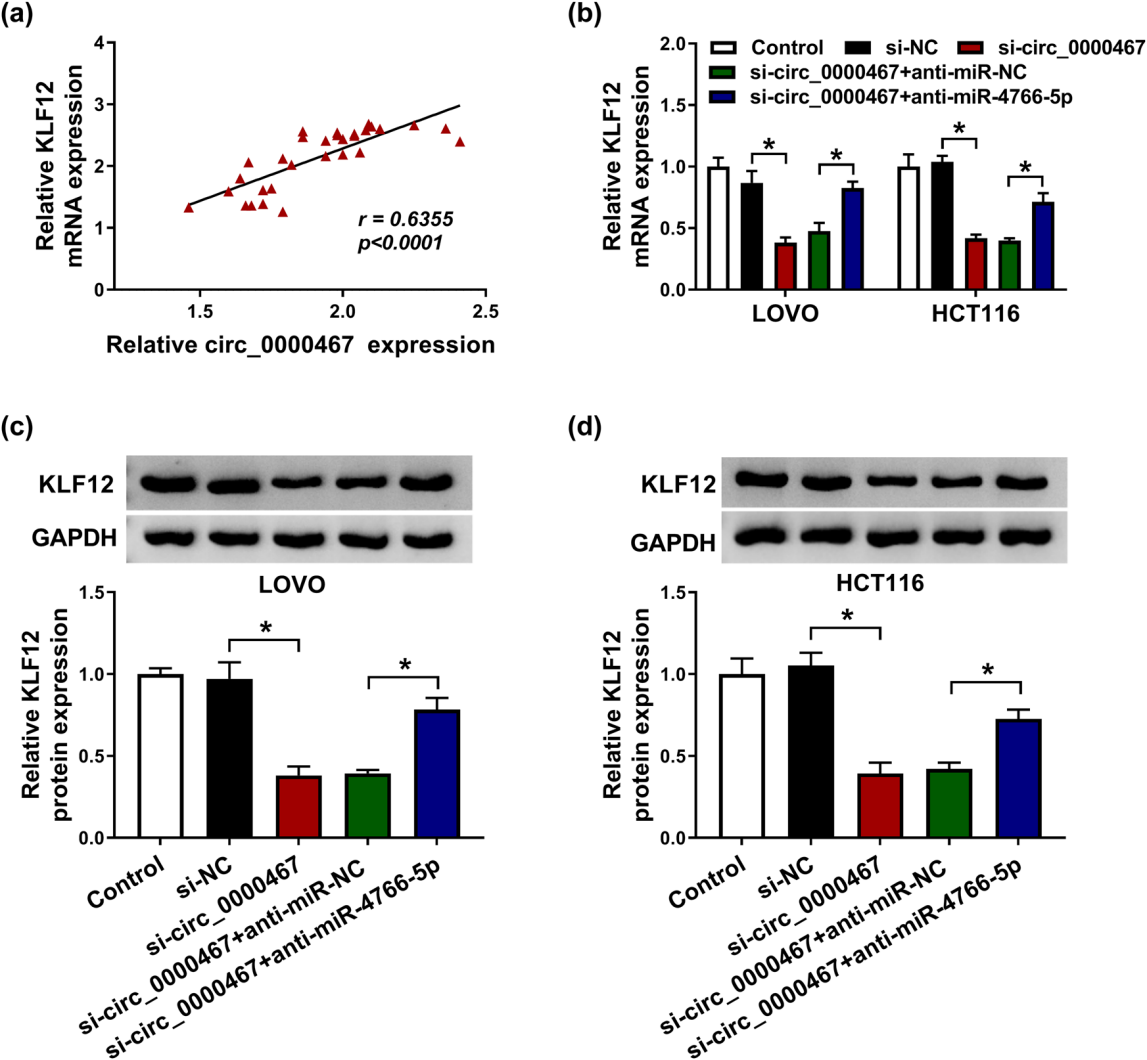
Silencing circ_0000467 repressed KLF12 expression via upregulating miR-4766-5p. (a) Pearson’s correlation coefficient was used for analyzing the relationship between circ_0000467 and KLF12 in CRC tissues. (b) The KLF12 mRNA level was examined by RT-qPCR in LOVO and HCT116 cells transfected with si-circ_0000467, si-circ_0000467 + anti-miR-4766-5p, or the relevant controls. (c and d) KLF12 protein expression was detected by WB in transfected LOVO and HCT116 cells. *P < 0.05.

Pearson’s correlation coefficient was used to analyze the relationship between circ_0000467 and KLF12 and the result indicated that there was a positive correlation (*r* = 0.6355, *P* < 0.0001) (Figure 7a). The expression analysis for KLF12 was performed in LOVO and HCT116 cells transfected with si-NC, si-circ_0000467, si-circ_0000467 + anti-miR-NC, or si-circ_0000467 + anti-miR-4766-5p. The data showed that the KLF12 mRNA (Figure 7b) and protein (Figure 7c and d) levels were downregulated in the si-circ_0000467 transfection group, while anti-miR-4766-5p transfection returned this inhibition. Therefore, the function of circ_0000467 in CRC was related to the regulation of the miR-4766-5p/KLF12 axis.

### 3.8 circ_0000467 downregulation retarded tumorigenesis of CRC *in vivo* via the miR-4766-5p/KLF12 axis

**Figure 8:**
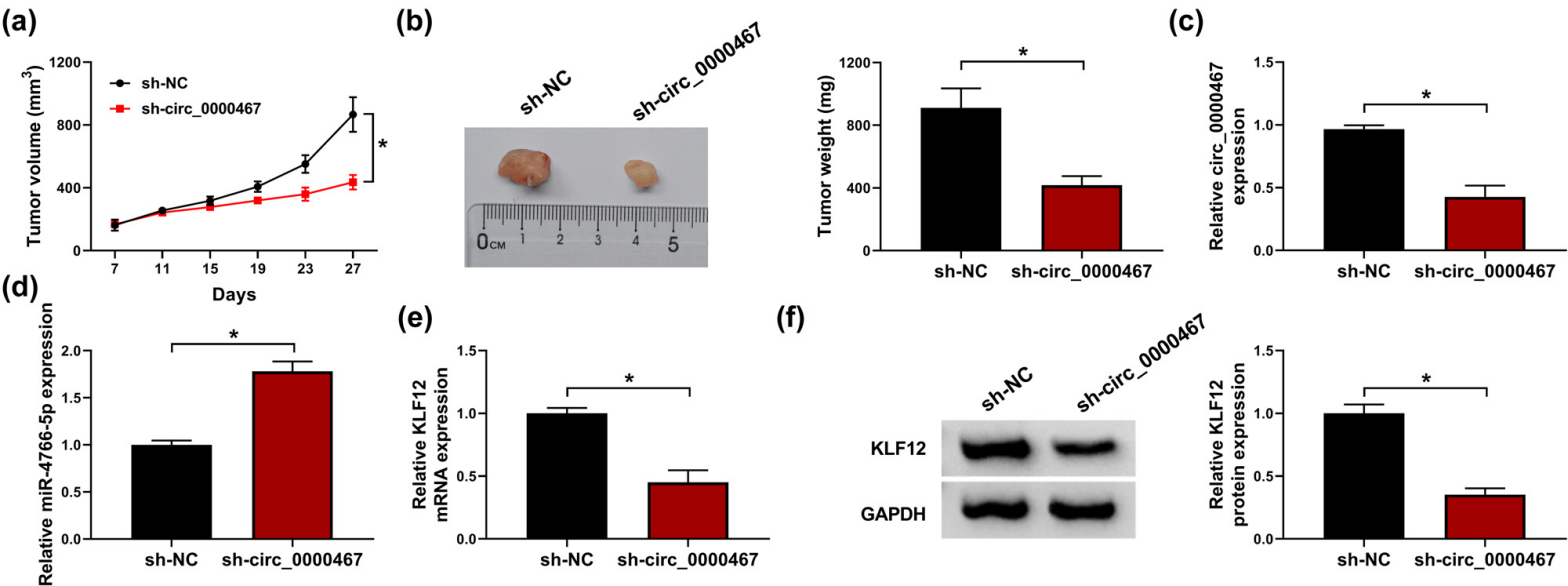
circ_0000467 downregulation retarded tumorigenesis of CRC *in vivo* via the miR-4766-5p/KLF12 axis. (a) Tumor volume of sh-NC or sh-circ_0000467 group was measured every 4 days after cell inoculation for 1 week. (b) Tumor tissues were weighed on an electronic scale. (c and d) The RT-qPCR was conducted to examine the expression of circ_0000467 and miR-4766-5p in tissues. (e and f) KLF12 mRNA (e) and protein (f) levels were tested using RT-qPCR and WB. *P < 0.05.

In the xenograft models of mice, we observed that tumor volume was lower in the sh-circ_0000467 group than in the sh-NC group (Figure 8a). After tumors were excised from the mice, the tumor weight of the sh-circ_0000467 group was also found to be reduced compared to the sh-NC group (Figure 8b). RT-qPCR analysis showed that sh-circ_0000467 successfully induced the inhibition of circ_0000467 level (Figure 8c). Moreover, the knockdown of circ_0000467 enhanced miR-4766-5p level (Figure 8d) but generated the opposite effect on KLF12 mRNA (Figure 8e) and protein (Figure 8f) levels. Altogether, it was found that circ_0000467 downregulation acted on the miR-4766-5p/KLF12 axis to inhibit tumorigenesis *in vivo*.

## 4 Discussion

CRC is a common cancer that causes huge physical and economic stress on patients. Seeking credible biomarkers and knowing the pathological mechanism of CRC are essential. In our report, we found that circ_0000467 was aberrantly expressed in CRC and promoted CRC progression by targeting the miR-4766-5p/KLF12 axis.

circRNAs have stable closed-loop structures and participate in the tumor process as inhibitors or promoters [18]. For instance, Li et al. attested that circMTO1 blocked cell metastasis in human bladder cancer [19]; Gao et al. reported that circ_0007059 suppressed proliferation and epithelial–mesenchymal transition of lung cancer cells [20]. circ_0000285 was overexpressed in cervical cancer, and promoted cell growth and metastasis [21]. circ_103809 also functioned as a carcinogenic factor of lung cancer *in vitro* and *in vivo* [22]. The circ_0000467 level was significantly increased in CRC tissues and cells. CRC cell proliferation, migration, invasion, and angiogenesis were all restrained but cell apoptosis was enhanced by the knockdown of circ_0000467. WB also manifested that pro-proliferative cyclinD1 was downregulated but pro-apoptotic cleaved-casp-3 and anti-metastatic E-cadherin increased as a result of circ_0000467 inhibition. Chen et al. found that circ_0000467 was an upregulated circRNA in GC and it had high prognostic value for GC patients [23]. Lu et al. found that the downregulation of circ_0000467 suppressed cell proliferation, migration, and invasion of GC cells [24]. In accordance with that in GC, we validated that circ_0000467 also served as an oncogene in CRC. Additionally, a recent study reported that circ_0000467 exerted inhibitory effects on CRC cell growth and metastasis by regulating the miR-382-5p/EN2 axis [25]. Our experimental analysis on the role of circ_0000467 in CRC progression was consistent with the findings of the research carried out by Xie and Pan [25]. However, the other potential regulatory mechanisms for circ_0000467 in CRC remain to be explored.

Previous studies have indicated that miR-4766-5p functioned as a tumor inhibitor in cancers such as GC and breast cancer [8,12]. Overexpression of miR-4766-5p also reduced cell viability, migration, and invasion while it enhanced cell apoptosis in hepatocellular carcinoma [26]. Here, our functional assay for miR-4766-5p affirmed that it inhibited CRC cell proliferation and metastasis. Zhan et al. stated that LINC00858 could act as a miR-4766-5p sponge to facilitate the progression of CRC by upregulating PAK2 level [9]. It might be interesting to investigate the potential of miR-4766-5p as a target of circRNA in CRC.

Indeed, the “miRNA sponge” is a common functional mechanism of circRNAs in most cancers [27]. For example, circ_0001982 worked as a miR-143 sponge to promote the carcinogenesis of breast cancer cells [28]. Cancer progression was promoted by the circ-cTFRC/miR-107 axis in bladder cancer [29] and the circHIPK3/miR-124 axis in gallbladder cancer [30]. In this study, miR-4766-5p was proved as a target of circ_0000467. In addition, the reverted assays demonstrated that the function of circ_0000467 was achieved by the miRNA sponge effect on miR-4766-5p. Moreover, miR-4766-5p could bind to KLF12 to induce the direct inhibition of KLF12 expression. miR-4766-5p impeded the developing process of tumors by regulating different targets, including SIRT1 in breast cancer and NKAP in GC [8,12]. In addition to PAK2 [9], KLF12 was found as a novel target for miR-4766-5p in CRC. The suppressive regulation of miR-4766-5p depended on targeting KLF12 in CRC.

Furthermore, our experimental data revealed that KLF12 expression was affected by circ_0000467 via sponging miR-4766-5p in CRC cells. KLF12 has been reported to be responsible for the function of circRNA via miRNA in cancer regulation. Guan et al. showed that circ_NOTCH3 contributed to the development and progression of basal-like breast carcinoma by competing with miR-205 to elevate KLF12 expression [31]. Chen et al. declared that circNEIL3 sponged miR-137 to promote the level of KLF12, thus accelerating cell proliferation in cervical cancer [32]. Also, circ_0003496 enhanced tumorigenesis and chemoresistance in osteosarcoma by regulating KLF12 via absorbing miR-370 [33]. Therefore, we concluded that the effect of circ_0000467 on CRC progression was partly associated with the KLF12 level via targeting miR-4766-5p. The results *in vivo* also confirmed the promoting regulation of circ_0000467 on tumorigenesis by affecting the miR-4766-5p/KLF12 axis.

**Figure 9:**
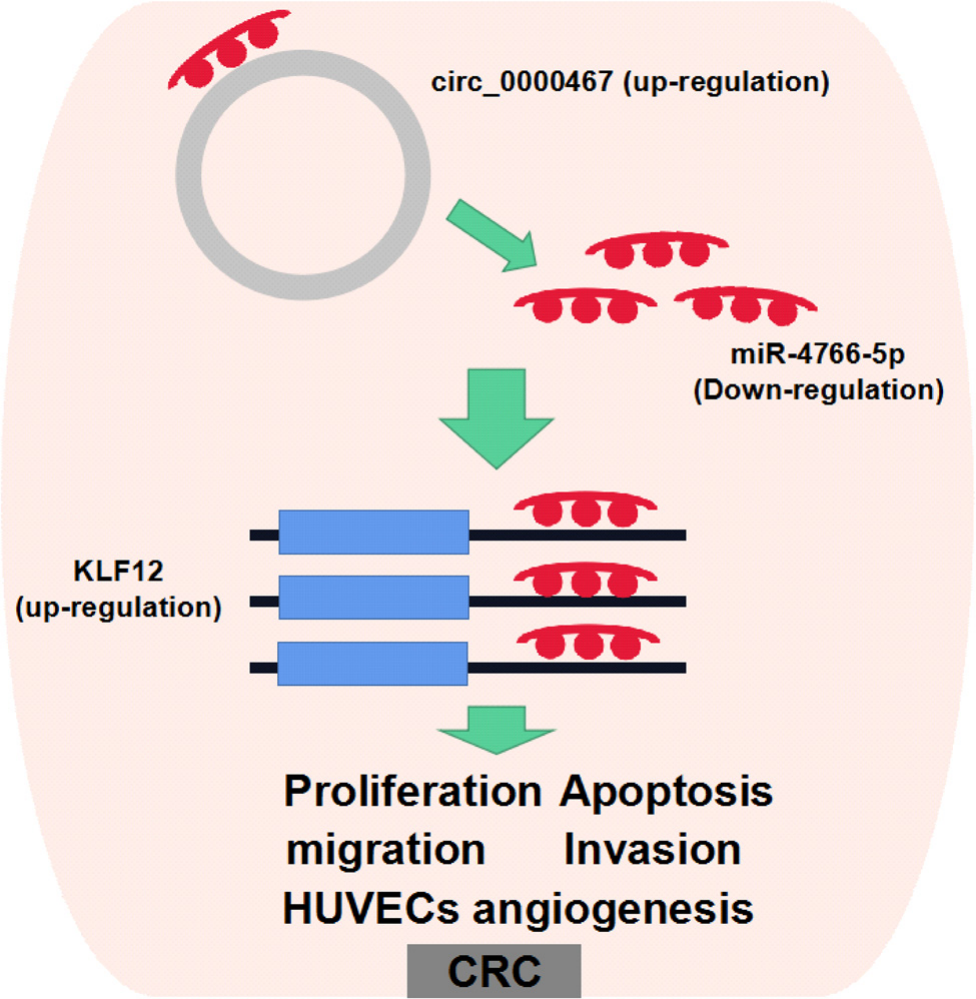
circ_0000467 sponged miR-4766-5p to upregulate KLF12 expression to regulate the cellular behaviors of CRC cells.

To sum up, this study suggested that circ_0000467 interacted with miR-4766-5p to promote the expression of KLF12 to evoke the malignant behaviors of CRC cells including the enhancement of cell proliferation, migration/invasion, angiogenesis, and the inhibition of cell apoptosis (Figure 9). The circ_0000467/miR-4766-5p/KLF12 signal axis was a novel mechanism for CRC molecular pathology. circ_0000467 may be available for the diagnosis and treatment of CRC.
